# In the presence of non-neutralising maternally derived antibodies, intradermal and intramuscular vaccination with a modified live vaccine against porcine reproductive and respiratory syndrome virus 1 (PRRSV-1) induce similar levels of neutralising antibodies or interferon-gamma secreting cells

**DOI:** 10.1186/s40813-022-00289-4

**Published:** 2022-11-04

**Authors:** Laia Aguirre, Yanli Li, Massimiliano Baratelli, Gerard Martín-Valls, Martí Cortey, Joel Miranda, Marga Martín, Enric Mateu

**Affiliations:** 1 Departament de Sanitat i Anatomia Animals, Universitat Autònoma de Barcelona (UAB), 08193 Cerdanyola del Vallès, Spain; 2 HIPRA, Amer (Girona), Girona, Spain

**Keywords:** Porcine reproductive and respiratory syndrome virus, Vaccine, Intramuscular, Intradermal, Maternally derived antibodies

## Abstract

The purpose of this study was to compare the immune response generated by the intramuscular and the intradermal vaccination route against the porcine reproductive and respiratory syndrome virus (PRRSV). Piglets from a seronegative and a seropositive farm were selected (n = 28 piglets per farm), and each group was divided into two groups and vaccinated after weaning with modified live vaccine Unistrain® PRRS (Laboratorios Hipra Amer, Spain) by the intramuscular (IM) or the intradermic (ID) route. For the following 6 weeks, animals were weekly bled to assess the humoral response by PRRSV-specific antibody ELISA and viral neutralisation test. At 0-, 3-, 4- and 6 weeks post-vaccination, peripheral mononuclear blood cells (PBMC) from eight animals per group were recovered to analyse cellular response by IFN-γ ELISPOT and lymphoproliferation. Serum IL-12 was also quantified by ELISA. Seroconversion was first detected 14 days post-vaccination (dpv) for both IM and ID routes, and peaked at 35 dpv (both IM groups and ID seropositive) or 42 dpv (ID seronegative). At 3 weeks after vaccination, 6/27 (22.22%) animals from negative origin had not seroconverted, and neutralising titres were significantly lower at 35 dpv compared to the seropositive origin (mean log_2_ titres of 1.36 and 4.25 respectively) Also, it was 10 times more probable for them to have high levels of IL-12 a week after vaccination than for animals of seropositive origin. Cellular immune response analysed by lymphoproliferation and IFN-γ ELISPOT was already present at 21 dpv and until 42 dpv, with no significant differences between groups except for a higher lymphoproliferation at 35 dpv in the IM seropositive group (Kruskal-Wallis, p < 0.05). These results indicate that the intradermal route induces an immune response equivalent to the classical intramuscular route even in presence of non-neutralising maternal immunity, which in this study has proven to facilitate seroconversion after vaccination with an heterologous strain.

**Supplementary Information:**

The online version contains supplementary material available at 10.1186/s40813-022-00289-4.

## Background

The porcine reproductive and respiratory syndrome (PRRS) is one of the main causes of economic loss in swine worldwide. Control of the infection is usually done by a combination of virus monitoring, biosecurity restrictions, herd management measures and vaccination. Although vaccination most often targets sows, piglet vaccination is increasingly being considered to protect weaners in farms that have endemic problems in nurseries.

Vaccination of piglets against PRRS virus (PRRSV) is usually performed during suckling or immediately after weaning. In endemically infected farms, most piglets present high levels of maternally derived antibodies (MDA) against PRRSV which may interfere with the development of an active immunity after vaccination [1, 2]. The general mechanisms of blocking by MDA for viruses are diverse (reviewed by Niewiesk [3]) and most often do not involve neutralization of the antigen but the interaction of MDA with B-cell receptors. However, the exact mechanism behind maternal blocking in PRRS is not fully elucidated and might involve neutralization as well.

Different strategies can be used to avoid the eventual interference caused by maternal antibodies on vaccine immunisation. For instance, one common approach is to administer repeated doses of the vaccines (commonly two doses separated by a 3-4-week period) close to the age at which MDA are expected to wane to compensate for the eventual detrimental effect of the MDA on the first dose. Another option is to deliver the antigen to anatomical sites where the concentration of MDA is lower than the concentration in the muscle so there is less chance of interference with the vaccination. The intradermal (ID) route has several advantages such as the reduction of the antigen needed to induce an immune response [4] and the potential of targeting a higher diversity of dendritic cells (reviewed by Combadiere and Liard [5]). Of special interest are Langerhans cells, that are particularly able to induce cytotoxic T cells [13] but are less efficient in inducing responses from B cells. On the other hand, in humans, CD14^+^ dermal dendritic cells are especially able to prime naïve B cells. This diversity of antigen-presenting cells in the skin can be advantageous for achieving effective immune responses after vaccination even when reduced amounts of antigen are available. Several studies proved that reduced doses of dermally-delivered antigens are effective for immunizing children with MDA against poliovirus [12, 14].

In PRRS, the immune response of vaccination by ID route has been proven to be similar to the intramuscular (IM) route [6–9] when pigs were not having specific MDA. However, much less is known about the efficacy of the ID route in the presence of MDA. The main objective of the present study was to compare the immune responses generated by a PRRSV live attenuated vaccine administered by either the ID or the IM routes in piglets with high levels of specific MDA or in seronegative piglets. For this purpose, 4-week old piglets from a seropositive and a seronegative farm were vaccinated with an heterologous strain from the one used in the farm, by either ID or IM route and weekly followed until 10 weeks of age. PRRSV-specific antibody ELISA and viral neutralisation test were done to assess humoral response, and cellular response was tested by IFN-γ ELISPOT and lymphoproliferation. Innate immunity was measured by ELISA of cytokines with anti-inflammatory (IL-10), pro-inflammatory (IL-12) or anti-viral (IFN-α) role.

## Results

### Clinical follow-up

No adverse reactions to vaccination were recorded. Two animals from the seropositive group died prior to vaccination, one because of an internal haemorrhage (probably because of a fight) and the other one because of diarrhoea. One additional animal from group ID-POS was withdrawn from the study on arrival at the experimental facilities because of a lameness produced during the load and transport. At the beginning of the study, there were 12 animals in ID-POS, 13 in IM-POS and 14 animals in IM-NEG and ID-NEG. During the study, three animals were euthanised because of the development of bacterial meningitis (two from ID-NEG and one from IM-NEG), and a piglet from the IM-POS group was found dead, which necropsy was compatible with meningitis. No other incidents were observed in the rest of the animals during the 42-day follow-up.

### Vaccine-induced viremia

**Fig. 1:**
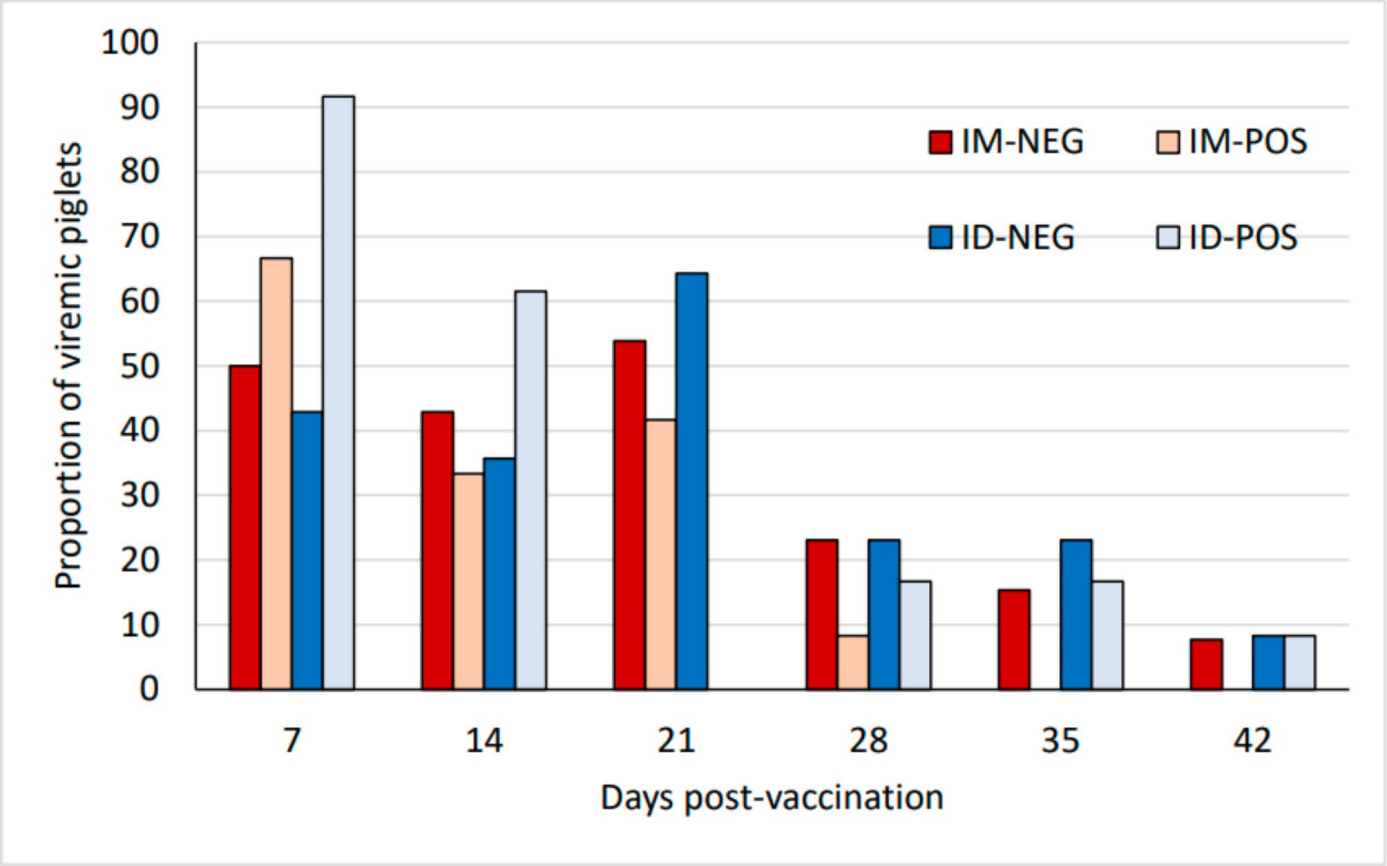
**Proportion of PCR-positive pigs for PRRSV at different time-points after vaccination.** Differences between groups were non-significant

Figure 1 shows the evolution of vaccine-induced viremia in the different groups. After vaccination animals were tested weekly to evaluate the presence of PRRS virus in blood. In all groups, vaccination produced a viremia at 7 days post-vaccination (dpv) (from 43 to 91% of the vaccinated animals, depending on the group). After 21 dpv the proportion of viremic animals dropped significantly and after 35 dpv, only 1 or 2 animals/group were positive. No significant differences between groups were observed regarding the proportion of positive animals. Regarding Ct-values, significant differences were observed at 7 dpv between ID-POS and the IM-NEG with lower Ct values for the latter (Supplementary material S1).

### Development of antibodies

The cohort of 2-week old pigs tested in the seropositive herd (n = 132) showed S/P values (ELISA) ranging between 0.44 and 2.24 (Supplementary material S2). The 28 selected animals had S/P values from 1.45 to 2.24. Those S/P values were in the quartile 75% of the distribution. At 4 weeks of age, when the vaccination was performed, animals in subgroup IM-POS had an S/P of 1.22 ± 0.28 versus 1.16 ± 0.23 for ID-POS (non-significant differences). As expected, animals in group NEG were seronegative at the moment of vaccination.

**Fig. 2:**
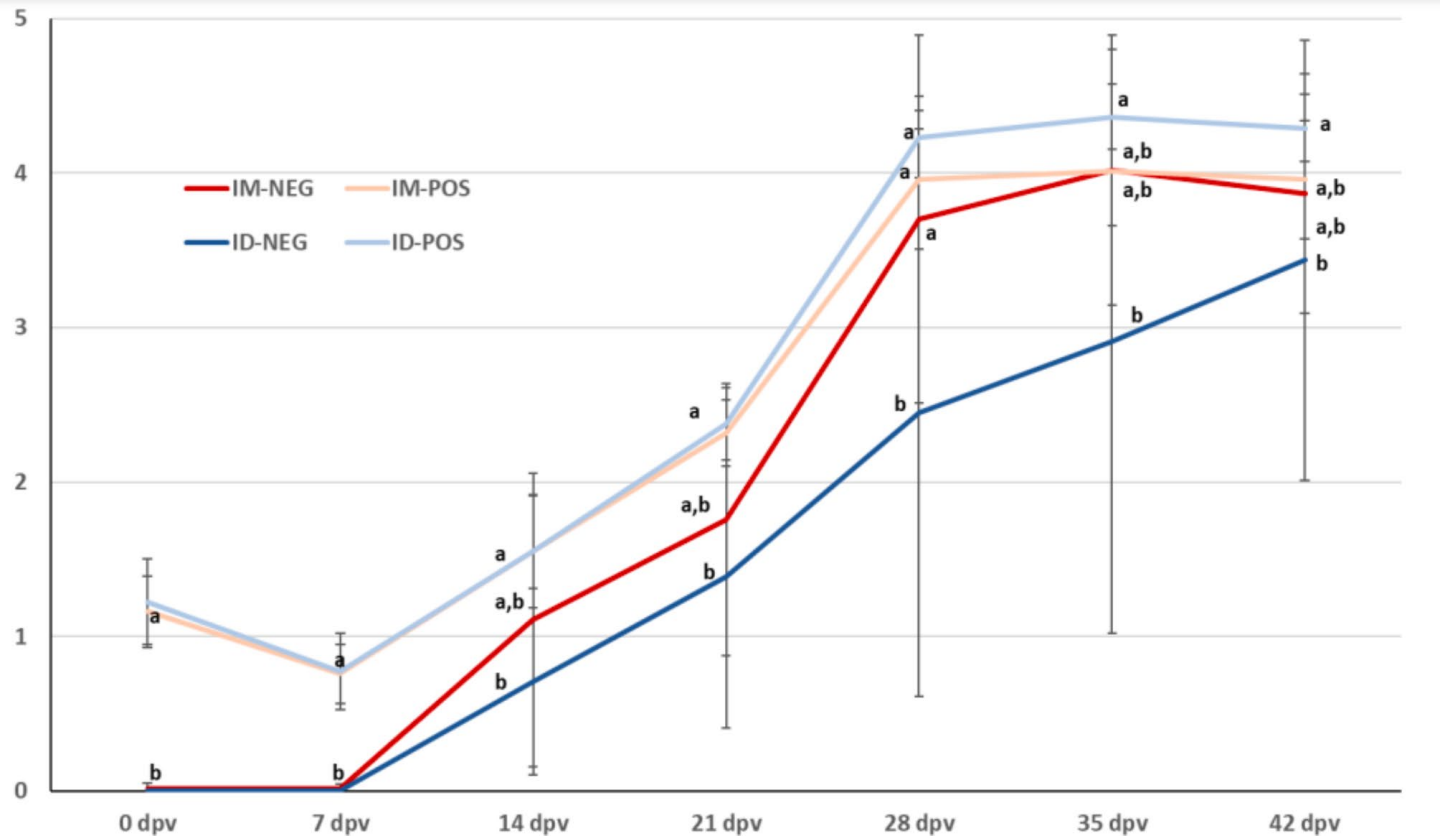
**Evolution of the S/P ratios in the different groups as determined by ELISA.** Statistically significant differences are indicated by different superscript letters

Figure 2 shows the evolution of average S/P values per group after vaccination. Vaccination produced a clear seroconversion in the seronegative animals (group NEG) that was noticeable by 14 dpv. Average S/P values increased until 35 dpv in the IM-NEG group, and until 42 dpv in the ID-NEG group. In the POS groups seroconversion was also observed, with increasing S/P ratios until day 28 dpv. It is worth noting that the ID-POS group presented the highest average S/P value at the end of the study, significantly different from that of the ID-NEG group. Differences between POS and NEG IM-vaccinated animals were non-significant.

**Fig. 3:**
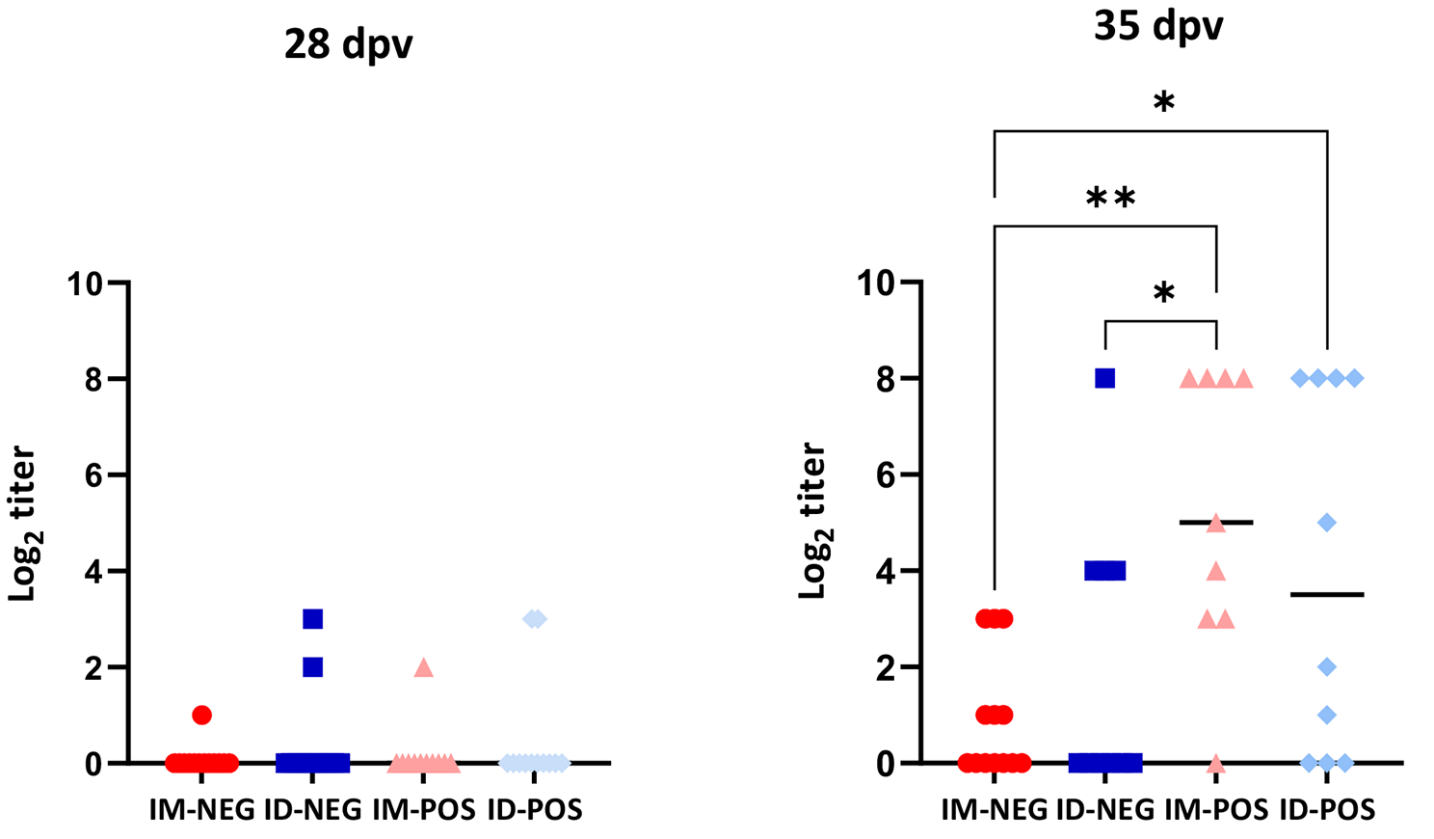
**Distribution of virus neutralisation titres in the different groups.** Only statistically significant differences (Kruskal-Wallis, p < 0.05 are indicated, * p < 0.05; ** p < 0.01). Each dot represents one examined individual

Sera obtained on the day of vaccination could neutralise the vaccine used in sows (titres of 2.75 ± 1.42 log_2_ and 3.2 ± 2.1 log2 in IM and ID, respectively, non-significant) but they were devoid of neutralisation capacity against the vaccine administered to piglets. The development of neutralising antibody titres induced by the administration of the vaccine was firstly detected at 28 dpv. At 35 dpv, significant differences were noticed between the groups that were vaccinated in the presence of MDA and the seronegative ones (Fig. 3, p < 0.05), with a mean titre of 4.25 log_2_ and 1.36 log_2_ respectively. The main difference was in the IM vaccinated groups, in which animals of the seropositive origin presented significantly higher titres compared to animals from the negative origin (Fig. 3, p < 0.01).

### Lymphoproliferation

**Fig. 4:**
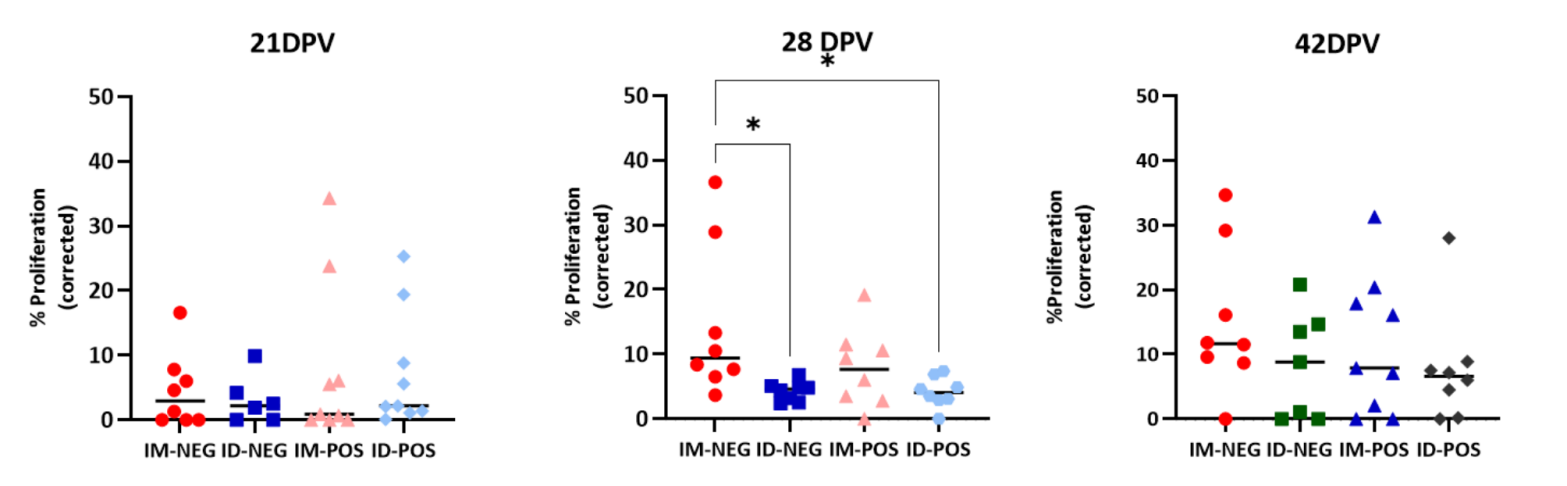
**Results of the proliferation assays.** The graphs depict the proportion of proliferating PBMC in cell cultures stimulated with the vaccine virus (21, 28 and 42 dpv, from left to right) at multiplicity of infection 0.1 once the spontaneous proliferation in mock-stimulated cultures was subtracted. Asterisks indicate the statistically significant differences (p < 0.05). Each dot represents an individual

PRRS-specific lymphocyte proliferation was clear by 21 dpv in some animals, which was more evident at 28 dpv and present in most animals at 35 dpv. Some differences in the proliferative responses were noticed at 35 dpv favouring the IM-NEG group (Kruskal-Wallis, p < 0.05), but they disappeared at 42 dpv (Fig. 4).

### IFN-γ secreting cells

**Fig. 5:**
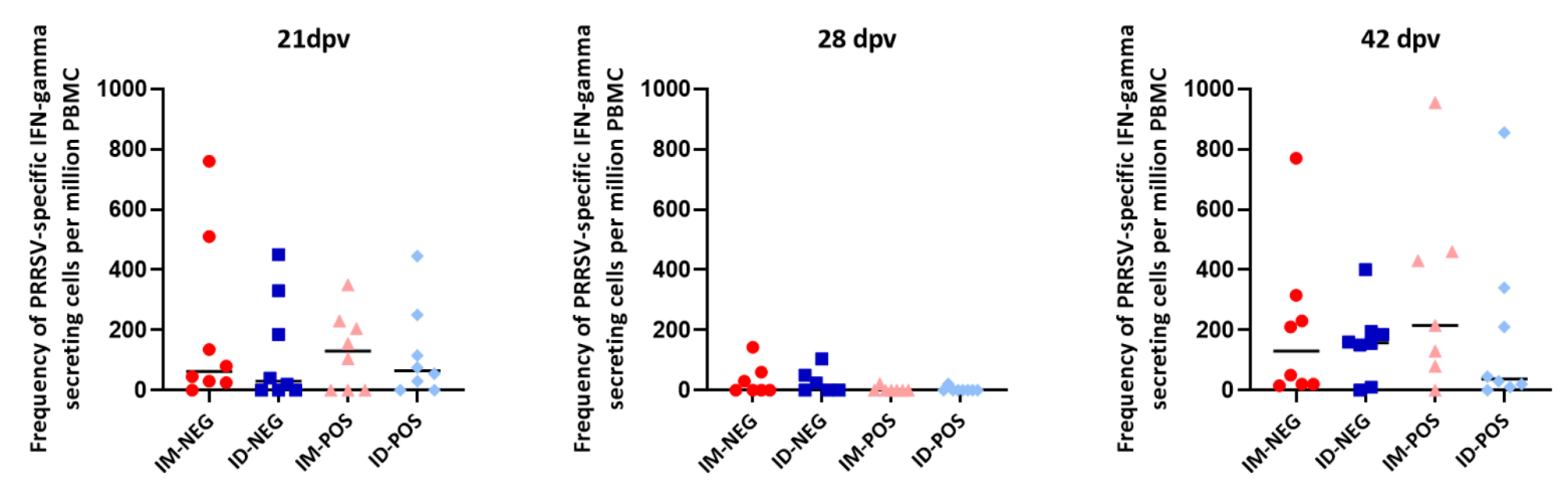
**Evolution of the virus-specific IFN-γ responses as determined by ELISPOT.** The graph depicts the frequencies of virus-specific IFN-γ secreting cells at 21, 28 and 42 dpv for the different groups. The bar indicates the median of each group. Differences between groups were non-significant. Each dot represents an individual

ELISPOT results were negative for all the animals at 0 dpv. Virus-specific responses were detected at 21 dpv in several animals from different groups and peaked at 42 dpv, with no significant differences between groups at any age (Fig. 5).

### Cytokine production

*IFN-α.* Levels of IFN-α in serum at 7 dpv ranged between 300 and 700 pg/mL, with no significant differences between groups (IM-NEG: 435 ± 83 pg/ml, ID-NEG: 420 ± 79; ID-POS: 454 ± 86; ID-POS: 481 ± 86).

*IL-10.* At 7 dpv, levels of IL-10 were undetectable or very low (< 64 pg/ml) in all animals with no differences between groups. At 14 dpv values higher than 100 pg/ml of IL10 could be detected only in sera of NEG animals, 4 in the ID subgroup (164-1,849 pg/ml) and 2 in the IM subgroup (106–553 pg/ml). Of these, at 21 dpv 2 animals still showed elevated IL10 levels in the ID-NEG group (249–505 pg/ml) and one in the IM-NEG group (273 pg/ml). In culture supernatants, IL-10 concentration was very low and with no significant differences.

*IL-12.* At 7 dpv it was 10 times more likely to be IL-12 positive (9/28 vs. 1/23; p = 0.033) for animals of the seronegative origin compared to the seropositive ones. At 14 dpv, all animals but three showed high levels of IL-12 in serum (195.1-3,821.6 pg/ml). Interestingly, of the 10 animals having the highest IL-12 levels at 14 dpv, 6 were the ones having elevated IL-10 at the same sampling time.

## Discussion

Interference of MDA with vaccination is a major concern when young piglets are to be vaccinated. In PRRSV endemic farms, circulation of the virus often starts in the farrowing units because of the existence of vertical transmission from sows to newborns, or very soon after weaning since MDA wane between 4 and 5 weeks of age (reviewed by Pileri and Mateu [10]). Given the fast spread of the virus in naïve populations, vaccination of piglets must be administered at weaning or even before, implying that most vaccinated piglets will have MDA.

The intradermal administration of vaccines is an increasingly interesting alternative for vaccination, being less invasive and more respectful of animal welfare [11], and that could diminish interference with MDA as shown for the human poliovirus vaccine administered to children [12]. The skin has a rich diversity of antigen-presenting cells that are highly efficient for capturing and transporting antigens to the draining lymph nodes. In the case of PRRSV, the intradermal administration of MLV PRRSV vaccines has proven to be as effective as the IM administration in naïve MDA-free pigs [6–9, 15]. The vaccine tested in the present study showed that both routes were equally effective in developing immunity against PRRS in absence of MDA, which is in line with what showed by the above authors; moreover, the vaccine was demonstrated to be able to produce similar levels of immunity by both routes even in presence of high maternal non-neutralising antibodies.

The mechanisms by which MDA interference occurs are not fully elucidated. Renson et al. [1] showed that homologous neutralising MDA titres of about 1:10 may produce some interference with the development of immunity in piglets. However, although neutralising antibodies (NA) may have a role in blocking the replication of the MLV virus, there is strong evidence in other viruses like measles virus favouring the hypothesis that blocking by MDA may involve the inhibition of B-cell responses by the cross-linking between B cell receptor with the Fcγ-receptor IIB by a vaccine–antibody complex, regardless of the neutralising capacity of the antibodies involved [3, 16, 17].

The design of the present study could have certainly overlooked the case of animals with high homologous NA titres, since the scenario selected was that where seropositive piglets did not have NA against the vaccine virus. Since NA against PRRSV usually have a narrow breadth of neutralisation [18], the capability of MDA to neutralise a particular vaccine virus will depend on what vaccine was used in the sows and on their previous contact with other PRRSV strains. Therefore, piglets being vaccinated with an heterologous strain is not an unsual scenario and would account for situations that can be encountered in the field, including the vaccination of the offspring of sows vaccinated with a different vaccine, or simply, of seropositive unvaccinated sows. The interference of MDA antibodies resulting from vaccination with different vaccines should be evaluated in the future.

The most remarkable results of the present study were on these animals of seropositive origin, which presented a stronger humoral response despite having high levels of MDA. This was clear for both S/P values (particularly for ID-POS) and virus NA titres of IM-POS group, significantly higher than IM-NEG. This phenomenon of increased response to vaccination in individuals with sub-neutralising levels of antibodies has been proven in humans that were vaccinated with an experimental attenuated vaccine against the dengue virus [19, 20]. In that case, the greater immune response in individuals with antibodies was attributed to increased infection of macrophages mediated by virus-antibody complexes. In the present case, Ct values of POS animals at 7 dpv were similar or higher to those of NEG animals. However, it is worth noting that there were more PCR-positive pigs at 7 dpv in the POS (IM and ID) compared to the NEG groups. Nevertheless, it has to be stressed that the different genetics in POS and NEG farms (DanBred x Pietrain and Duroc x Landrace, respectively) might have had an impact on the immune response.

Regarding the cell-mediated responses, the presence of MDA did not significantly interfere with the development of proliferative or IFN-γ responses. In other studies, it has been shown that vaccine blocking by MDA usually affects the development of humoral responses but not the development of cell-mediated immunity [21, 22].

In the present case, the development of vaccine-induced viremia was similar in all groups following a pattern described in other previous works [1, 23]. As shown before, some animals did not develop detectable viremia while others persisted as positive for several weeks. One possible element involved in this different behaviour could have been the IFN-α response. Renson et al. [1] reported a potential correlation of this phenomenon with decreased response to the vaccine. This was not our case since all animals had comparable levels of this cytokine in serum.

Interestingly, the results showed that seronegative animals had a higher probability of having high levels of IL-12 in serum after vaccination than seropositive ones. This suggests a differential targeting of TLRs in animals with or without antibodies against the vaccine virus. Most of the animals with the highest IL-12 levels in serum were the ones with high IL-10 levels. A possible explanation could be in the homeostatic action of IL-10 to counterbalance excessive IL-12 production. It is known that IL-10 is a potent regulator of IL-12 transcription [24].

These results raise questions concerning the role of MDA in PRRS vaccination. The present results would indicate that in cases where the MDA were subneutralising (i.e., heterologous vaccines in sows and piglets), humoral immunity induced by MLV vaccination could even be enhanced. Further research should be done in this area to figure out what were the mechanisms leading to this difference including the possible enhancement of the vaccine virus replication and also whether vaccine-antibody complexes result in different stimulation of antigen-presenting cells.

## Conclusion

In conclusion, intradermal and intramuscular vaccinations have been proven as equivalent administration routes for the immunity parameters evaluated in this experimental study. The presence of non-neutralising antibodies at the time of vaccination resulted in enhanced humoral response without being detrimental to the development of cell-mediated responses.

## Materials and methods

### Animals and experimental design

The study was approved by the Committee for Ethics in Human and Animal Experimentation of the Universitat Autònoma de Barcelona (number CEEAH 5357) and by the Generalitat de Catalunya (FUE-2020-01836411).

Pigs were recruited from two different herds; in the first, high levels of PRRSV-specific non-neutralising MDA were detected (group POS) whereas in the second no PRRSV antibodies were detected (group NEG). Group POS were DanBred x Pietrain crossbred animals. The farm was positive stable according to the revised AASV scheme of classification as PRRSV was not detected at weaning [25]. Sows were vaccinated with Porcilis™ PRRS (MSD Animal Health) every 3 months, last vaccination was implemented approximately two months before farrowing. At two weeks of age, 132 piglets were randomly selected from the same farrowing batch and bled to determine the levels of MDA by ELISA (PRRS X3 Ab, Idexx). The absence of PRRSV circulation in the group was confirmed by RT-qPCR (VetMax PRRS EU&NA 2.0 RT-qPCR, ThermoFisher, Madrid, Spain). The 28 animals with the highest S/P ratios as determined by the test were selected for the experiment. Group NEG animals (n = 28) were Duroc x Landrace pigs obtained from a historically PRRSV-free farm and its status was confirmed as well by ELISA. In both cases, animals were weaned at 3 weeks of age and transported to the experimental facilities located in the UAB. There, they were ear-tagged and randomly divided into two subgroups that were housed in physically separated boxes. One subgroup was assigned the intramuscular (IM) vaccination and the other was assigned the intradermal (ID) vaccination. Thus, the final design contained 4 subgroups: IM-POS, IM-NEG, ID-POS, and ID-NEG according to the serological status and the route of administration of the vaccine. Pigs were left to acclimate for a week before the administration of the vaccine.

At 4 weeks of age, animals were bled and the PRRSV-1 vaccine was administered (Unistrain® PRRS, Laboratorios Hipra). The IM vaccination was performed by the injection of 2 ml of the vaccine in the neck muscles; the ID vaccination was performed by delivering 0.2 ml of the vaccine in the neck using a needle-free device (Hipradermic® 3.0). Each dose of the vaccine contained 1 × 10^4.3^ TCID_50_ as titrated in MARC-145 cells.

### Sampling and sample processing

Animals were monitored for 42 days after vaccination. Blood samples were obtained weekly from all animals to determine the development of PRRSV-specific antibodies and PRRSV vaccine viraemia. Heparinized blood samples were additionally taken from 8 animals/group at 0-, 21-, 28-, and 42 dpv. The heparinized samples were used to obtain peripheral mononuclear blood cells (PBMC) by gradient density centrifugation using Histopaque-1077® (Sigma-Aldrich) and Sepmate™ tubes (Stemcell Technologies, Saint-Égrève, France). The resulting PBMC were frozen in Cryostor® CS10 (Merck) and stored in liquid nitrogen until further analysis. PBMC were recovered as described elsewhere [26] and viability was checked by trypan blue staining (0.4%). Only samples with > 90% viability were used.

### Serological analysis

Serum samples were tested for the presence of PRRSV specific antibodies by HerdCheck® PRRS X3 Ab test Idexx ELISA.

Viral neutralisation tests were performed in sera from 0-, 21-, 28-, 35-, and 42 dpv using the vaccine strain. For group POS animals, samples of 0 dpv were also tested against the vaccine strain used for the sows in the origin farm (Porcilis). The neutralisation tests were performed following the procedure described by Yoon et al. [27] with minor modifications. Briefly, sera were inactivated at 56 °C for 30 min and diluted from 1:2 to 1:256 in Minimum Essential Medium with non-essential amino acids, sodium pyruvate, 100 IU/mL penicillin and 100 µg/mL streptomycin. Equal volumes (100 µl:100 µl) of the diluted serum and the vaccine strain adjusted at 2,000 TCID_50_/mL were mixed and incubated overnight at 4 °C. Then, 100 µL of the serum-virus mixture was transferred to MARC-145 monolayers in 96 well-plates. Negative (only medium) and virus controls (virus at working dilution and at 200, 20, and 2 TCID_50_/mL) were also included in the plate, as well as a negative and positive serum (post-vaccination serum of a sow) as controls. Plates were read after 6 days of incubation. The neutralisation titres were assessed by the development of the cytopathic effect and confirmed by immunofluorescence using the monoclonal antibody 1CH5 (Eurofins, Madrid). The neutralisation titre was the log_2_ of the reciprocal of the highest dilution without cytopathic effect and without significant fluorescence.

### Quantification of PRRSV RNA

Quantitative PCR for the quantification of PRRSV1 in serum was performed as previously reported [28]. RNA extraction was performed with the BioSprint 96 One-For-All-Vet kit (Qiagen), according to a BS96 Vet 100 protocol. Briefly, 100 µl serum was mixed with 300 µl RLT, 300 µl Isopropanol, 25 µl MagAttract Suspension G and 2.7 µl carrier RNA. Then, 700 µL mixed sample was applied to the S-Block plate. The kit BS96 Vet 100 protocol for RNA isolation was then followed. Finally, RNA was eluted in 80 µl nuclease-free water and the amount of PRRSV genome determined.

### Lymphoproliferation

Isolated PBMC were stained with CellTrace™ Violet (ThermoFisher) following the manufacturer’s instructions. Briefly, 1 × 10^5^ PBMC were dispensed on 96-well round-bottomed plates and the vaccine strain was added at a multiplicity of infection (MOI) of 0.1 (cultures were done in triplicate). In parallel, phytohemagglutinin-stimulated cultures (PHA, 10 µg/mL) or mock-stimulated cultures (RPMI medium) were used as positive and negative controls respectively. Plates were incubated for 5 days at 37 °C in a 5% CO_2_ atmosphere. Then, cell cultures were recovered and centrifuged at 300 g for 10 min. The resulting pellet was resuspended in PBS and analysed in a Cytoflex flow cytometer (Beckman Coulter, CA, USA). Flow cytometer files were analysed using the FCS Express Flow Cytometry 6 (de Novo Software). For each sample, the PRRSV-specific proliferation was calculated as follows: percentage of cell proliferation in the PRRSV stimulated cultures - percentage of cell proliferation in the negative control. A relative proliferation index was also calculated as the proportion of cell proliferation in PRRSV-stimulated culture versus the proportion of cell proliferation in the mock-stimulated cultures.

### IFN-γ ELISPOT

IFN-γ ELISPOT was performed as described by Zuckerman et al. [29] with minor modifications. Briefly, Costar 3590 plates (Corning) were coated with 50 µL of the monoclonal antibody P2G10 against porcine IFN-γ (ref. 559,961; BD Pharmingen, NJ, USA) at 5 µg/mL diluted in carbonate-bicarbonate buffer (0.15 M pH 9.5). After overnight incubation at 4 °C, plates were washed five times with PBS and blocked for 1 h with RPMI containing 10% foetal calf serum. Following the removal of the blocking solution, 50 µL/well of PBMC were dispensed at 1 × 10^5^ PBMC/well (negative control and virus stimulated wells) and 5 × 10^4^ PBMC/well (PHA-stimulated wells). PRRSV-stimulated wells were added to the vaccine strain at a MOI of 0.1. Mock-stimulated cultures (culture medium) and PHA-stimulated wells (10 µg/mL) were added as negative and positive controls, respectively. Plates were incubated overnight at 37 °C with 5% CO_2_. After washing, the biotinylated detection antibody P2C11 (ref. 559,958; BD Pharmingen) was added at 0.5 µg/mL diluted in PBS with 0.4% bovine serum albumin. After a 1 h incubation at 37 °C, 50 µL of streptavidin-peroxidase was added at 0.5 µg/mL (ThermoFisher) and the reaction was developed by adding insoluble TMB (50 µL/well). Spots were counted in a Leica stereoscope after a 10-minute incubation in the dark. Samples were tested in triplicate. The frequencies of responding cells were calculated by subtracting the counts in the mock-stimulated wells from the counts obtained in virus-stimulated wells for each sample. Results were expressed as the number of responding cells per 1 × 10^6^ PBMC.

### Cytokine ELISA in culture supernatant and serum

ELISA for cytokines IL-10, IL-12 and IFN-α were performed in PBMC culture supernatants and/or serum. Cell culture supernatants were obtained from PBMC cultures produced in the same conditions as the ELISPOT. Supernatants were collected after overnight incubation of the PBMC and frozen at − 80 °C until further analysis. Details of the samples analysed are shown in Table 1.

**Table 1:** Type and date of samples analysed for cytokines

Cytokine	Sample	Date
**IL-10**	Serum	7, 14, 21 dpv
	Culture supernatant	21 dpv
**IL-12**	Serum	7, 14, 21 dpv
**IFN-α**	Serum	7 dpv

Antibody pairs and kits used are shown in supplementary materials (S3). Optimal work concentrations of each antibody were previously determined using the reference standards provided by each kit. Cytokine concentrations in the supernatants were estimated by calculating a regression formula obtained from the results produced by a serial dilution of the standard provided by the kit.

### Statistical analysis

Data were analysed by StatsDirect v.3.3.5 and GraphPad 9.2 software. Differences between groups (continuous variables) were tested by ANOVA (Tukey for multiple comparisons). χ^2^ (2xK) was used to compare the proportion of responses from the different groups.

## Electronic Supplementary Material

Below is the link to the electronic supplementary material.

Supplementary Material 1

Supplementary Material 2

Supplementary Material 3

## Data Availability

The datasets used and/or analysed during the current study are available from the corresponding author on reasonable request.
