# Supplementary material for: In the presence of non-neutralising maternally derived antibodies, intradermal and intramuscular vaccination with a modified live vaccine against porcine reproductive and respiratory syndrome virus 1 (PRRSV-1) induce similar levels of neutralising antibodies or interferon-gamma secreting cells
Source: Porcine Health Manag. 2022 Nov 4;8:47. doi: 10.1186/s40813-022-00289-4 (PMC9636649; doi:10.1186/s40813-022-00289-4)
Supplement: Supplementary file 1 — Supplementary Material 1 [file 40813_2022_289_MOESM1_ESM.docx]

**Supplementary material S1. Average Ct values for the vaccine-induced viremia at 7dpv.** The graph depicts the average Ct-value of PCR-positive animals at 7 dpv (plus maximum, minimum, 25% and 75% quartiles). Different superscript letters indicated significant differences (p<0.05, Kruskal-Wallis test).

**c**

**b,c**

**b,c**

**a,b**
