# Supplementary material for: In the presence of non-neutralising maternally derived antibodies, intradermal and intramuscular vaccination with a modified live vaccine against porcine reproductive and respiratory syndrome virus 1 (PRRSV-1) induce similar levels of neutralising antibodies or interferon-gamma secreting cells
Source: Porcine Health Manag. 2022 Nov 4;8:47. doi: 10.1186/s40813-022-00289-4 (PMC9636649; doi:10.1186/s40813-022-00289-4)
Supplement: Supplementary file 2 — Supplementary Material 2 [file 40813_2022_289_MOESM2_ESM.docx]

**Supplementary material S2. Distribution of S/P ratios at the moment of the selection of seropositive animals.** The graph depicts the distribution of S/P ratios for the group of animals sampled at 2 weeks of age for the selection of POS individuals.

**
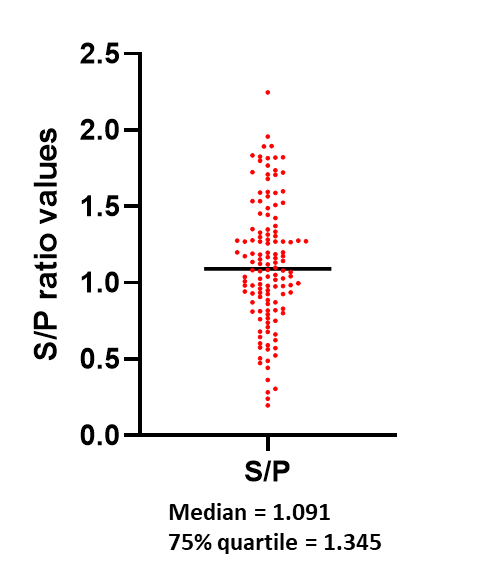
**
