# Supplementary material for: In the presence of non-neutralising maternally derived antibodies, intradermal and intramuscular vaccination with a modified live vaccine against porcine reproductive and respiratory syndrome virus 1 (PRRSV-1) induce similar levels of neutralising antibodies or interferon-gamma secreting cells
Source: Porcine Health Manag. 2022 Nov 4;8:47. doi: 10.1186/s40813-022-00289-4 (PMC9636649; doi:10.1186/s40813-022-00289-4)
Supplement: Supplementary file 3 — Supplementary Material 3 [file 40813_2022_289_MOESM3_ESM.docx]

**Supplementary material S3. Antibodies and kits used for cytokine ELISAs**

| **Cytokine** | **Antibodies/kit used – Refference – Supplier** |
| --- | --- |
| **IL-10** | Swine IL-10 Antibody Pair ELISA Kit (CSC0103, ThermoFisher) |
| **IL-12** | Porcine IL-12/IL-23 p40 (DY912, RD Systems, Minneapolis, USA) |
| **IFN-α** | Porcine IFN-alpha ELISA (ES7RB, ThermoFisher) |
